# pcaGoPromoter - An R Package for Biological and Regulatory Interpretation of Principal Components in Genome-Wide Gene Expression Data

**DOI:** 10.1371/journal.pone.0032394

**Published:** 2012-02-27

**Authors:** Morten Hansen, Thomas Alexander Gerds, Ole Haagen Nielsen, Jakob Benedict Seidelin, Jesper Thorvald Troelsen, Jørgen Olsen

**Affiliations:** 1 Department of Cellular & Molecular Medicine, The Panum Institute, University of Copenhagen, Copenhagen, Denmark; 2 Department of Biostatistics, University of Copenhagen, Copenhagen, Denmark; 3 Department of Gastroenterology, Medical Section, Herlev Hospital, University of Copenhagen, Copenhagen, Denmark; 4 Department of Science, Models and Systems, University of Roskilde, Roskilde, Denmark; University of Minnesota, United States of America

## Abstract

Analyzing data obtained from genome-wide gene expression experiments is challenging due to the quantity of variables, the need for multivariate analyses, and the demands of managing large amounts of data. Here we present the R package pcaGoPromoter, which facilitates the interpretation of genome-wide expression data and overcomes the aforementioned problems. In the first step, principal component analysis (PCA) is applied to survey any differences between experiments and possible groupings. The next step is the interpretation of the principal components with respect to both biological function and regulation by predicted transcription factor binding sites. The robustness of the results is evaluated using cross-validation, and illustrative plots of PCA scores and gene ontology terms are available. pcaGoPromoter works with any platform that uses gene symbols or Entrez IDs as probe identifiers. In addition, support for several popular Affymetrix GeneChip platforms is provided. To illustrate the features of the pcaGoPromoter package a serum stimulation experiment was performed and the genome-wide gene expression in the resulting samples was profiled using the Affymetrix Human Genome U133 Plus 2.0 chip. Array data were analyzed using pcaGoPromoter package tools, resulting in a clear separation of the experiments into three groups: controls, serum only and serum with inhibitor. Functional annotation of the axes in the PCA score plot showed the expected serum-promoted biological processes, e.g., cell cycle progression and the predicted involvement of expected transcription factors, including E2F. In addition, unexpected results, e.g., cholesterol synthesis in serum-depleted cells and NF-κB activation in inhibitor treated cells, were noted. In summary, the pcaGoPromoter R package provides a collection of tools for analyzing gene expression data. These tools give an overview of the input data via PCA, functional interpretation by gene ontology terms (biological processes), and an indication of the involvement of possible transcription factors.

## Introduction

Working with genome-wide gene expression data is challenging for the typical molecular biologist with training mainly focusing on laboratory techniques and only to lesser extend in the fields of mathematics or biostatistics. The large number of gene expression measurements available requires a meaningful reduction of the data set to make its results comprehensible. Data typically originate from DNA microarray hybridization experiments or, more recently, from next-generation sequencing experiments. An example of an experiment requiring genome-wide gene expression analysis is the extraction of RNA from a tissue sample taken *in situ* or from an *ex vivo* cultured cell line. The differences in mRNA levels between the different samples can be ascribed to three different effects: consequences of cellular signal transduction, cellular differentiation or the migration of cells into or out of the tissue. Under these circumstances, key transcription factors are responsible for establishing differences in the mRNA levels. Moreover, the transcription factors involved can often be linked to specific biological processes. For instance, the transcription factor NF-κβ is linked to inflammation [1], whereas the transcription factor HNF-4a is linked to lipid metabolism [2]. Therefore, data analysis of genome-wide gene expression data should allow for the interpretation of differences between groups of experiments in terms of transcription factor involvement and functional biological terms.

Several data analysis strategies for genome-wide gene expression data combine an unsupervised approach for reducing the dimension of the dataset with a supervised approach for drawing conclusions (for reviews see [3], [4], [5]). Along with the advent of DNA-microarray technology, cluster analysis has become a popular accompaniment of unsupervised investigations of high-dimensional data. Commonly used cluster analysis methods display gene expression data using heat maps and dendrograms [6], [7], [8]. Principal component analysis (PCA) and the related correspondence analysis (CA) represents another class of explorative unsupervised multivariate analysis methods that provide dimension reduction, and even though the method was first introduced into chemistry and biology in the late 1970's (for review see [9]), it was already described in the early twenties century [10]. The usefulness of PCA for analysis of genome-wide gene expression data has recently been reviewed [11]. However, whereas clusters of microarray hybridization experiments are typically easily distinguishable in standard PCA plots with few dimensions, the axes are not easily interpretable. We have previously demonstrated that PCA can provide an experiment-oriented view in combination with a functional interpretation of the PCA axes with respect to transcription factor involvement and biological function [12], [13], [14]. Although it is currently possible to link PCA with annotation analysis and overrepresentation analysis of predicted transcription factor binding sites, no software package available is designed to streamline this analysis strategy. It is necessary to use several software packages and to reformat the data between the different packages. Moreover, the bioconductor repository [15] holds at present 516 R packages, but none of these packages implement a transcription factor binding site overrepresentation analysis algorithm. Some of the bioconductor packages implement PCA (e.g. MADE4 [16] and pcaMethods [17]) and others annotation analysis (e.g. GOseq [18] and GOstats [19]), but these packages are not designed to work together. It was therefore the purpose of the present work to develop a single R package with a number of wrapper functions that would easily combine PCA with annotation analysis and transcription factor binding site overrepresentation analysis. Thus, the coupling of the intuitive understanding of differences between groups of experiments, the potential involvement of transcription factors and biological processes is automated by the pcaGoPromoter package. Compared with other commercial and open source pathway analysis software [20], the pcaGoPromoter is unique in using PCA score plot interpretations.

Currently, the package provides fast and straightforward data analysis for any genome-wide gene expression data platform using gene symbols or Entrez IDs as probe identifiers. In addition, several Affymetrix GeneChip platforms are also supported. In this work, we describe a serum stimulation experiment using human monocytes that was specifically designed to illustrate the use of the pcaGoPromoter package algorithms and tools.

## Materials and Methods

### Program description

The pcaGoPromoter package provides functions that have been designed for use with any gene expression analysis platform. In this report, however, we use data derived from the Affymetrix GeneChip platform for exemplification. The overall idea was to achieve an interpretation of the score plot axes of a PCA (function pca) in terms of biological processes (function GOtree) and the transcription factors involved (function primo).

A pcaGopromoter online version providing access to the most important plot functions is available at http://gastro.sund.ku.dk/brew/pcaGoPromoter.html.

### Data import

The pcaGoPromoter package supports Bioconductor's ExpressionSet class [15], however, in addition any normalized data can be used when formatted as a table with either Affymetrix probe set IDs, gene symbols or Entrez IDs as row names and experiment IDs as column identifiers. The serum stimulation data used as an example in the present work originated from the Affymetrix GeneChip platform, and the required pre-processing of the CEL files was performed with the affy package [21]. A data object was created with the ReadAffy function. Background correction and normalization was performed using the rma function [22]. The pca and GOtree functions work with any Affymetrix GeneChip, which is supported by Affymetrix CDF files, whereas the primo function comes with data files that support the most popular human (HG-U133 plus 2.0 and Human Gene ST 1.0), mouse (Mouse Genome 430 2.0 Array) and rat (Rat Genome 230 2.0 Array) GeneChip arrays. In addition the primoData function allows custom data files for primo to be produced by the user.

### Principal component analysis using the function pca

PCA is a well-established method for multivariate analyses [9], [23]. PCA reduces dimensionality by projecting experiments (each hybridization experiment) into a new subspace with fewer dimensions than the original space of the variables (in our case probe set IDs). It is important to note that PCA also can be used with the experiments as variables. pcaGoPromoter is, however, only intended for use in a setting with probes as variables. Each hybridization experiment yielded a vector of p expression levels **X_i_** referring to p probe set IDs on the chip. The data from n hybridization experiments were used to compute 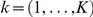 principal components:
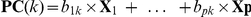
where 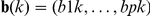 is a loading vector which satisfies the constraint 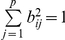. The first principal component **PC**(1) explains most of the variance of the data, the second principal component **PC**(2) second most, and so forth.

The loading b_pk_ quantifies the importance of the p′^th^ probe set ID on the chip for the k′^th^ dimension of the reduced predictor space. The sign and magnitude of the loadings were used to find important probe set IDs for functional interpretation.

In pcaGoPromoter, the function pca calculates the principal components of a data matrix with hybridization experiments in columns and probe set IDs in rows, by internally calling the function prcomp of the R base package ‘stats’. It should be noted that pca uses the transformed input matrix for calculations, as the convention for PCA is that experiment are in rows and variables in columns. The function getRankedProbeIds works on pca objects and is used to select the most important positive and negative probe set IDs based on their loadings. Going forward, we use a selection of the 2.5% probes set IDs with highest or lowest loadings, respectively, as an example. In a separate section under the Results and discussion section the selection of this parameter is discussed.

### Mapping principal component axes to enriched gene ontology terms using the function GOtree

We were interested in joining a functional interpretation to the directions of the axes for each principal component in the PCA score plot. The Gene Ontology (GO) Consortium [24] provides a set of databases that contain functional annotations for genes. The pcaGoPromoter package associates GO terms with biological processes for each principal component in both directions. This is done by calculating the overrepresentation of the GO terms in the annotation of genes with high absolute loadings for each principal component. This calculation is performed using the function GOtree, which operates either on the Affymetrix probe set IDs, gene symbols, or Entrez IDs. In case the input is not of class ExpressionSet, the input type is controlled with the argument inputType for the function GOtree. Objects obtained with the function GOtree are then visualized in a tree structure of overrepresented GO terms along with their corresponding p-values. The calculation for overrepresentation can be performed using either Fisher's exact test for proportions, or with an exact test for the number of successes in a Bernoulli sequence (controlled with the argument statisticalTest). The calculation details for overrepresentation of annotation terms in gene lists have earlier been published [25], [26].

### Mapping principal component axes to enriched promoter cis-elements using the functions primoData and primo

Transcription factors can be organized according to their DNA-binding motifs. The TRANSFAC database [27] and the Jaspar database [28] contain information about consensus DNA-binding motifs for a wide variety of transcription factors. Information about the binding sequences is organized in position weight matrices.

**Figure 1:**
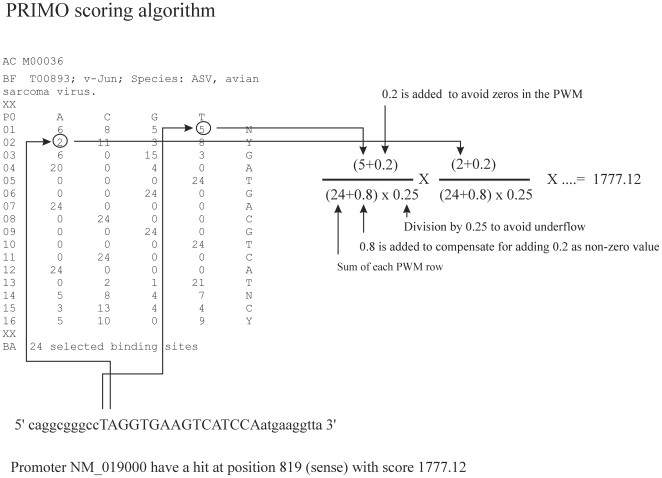
Description of the PRIMO algorithm. This figure shows the calculation of a position weight matrix (PWM) score for a specific DNA sequence. The sequence window under calculation is shown at the bottom in capital letters. To the left is the PWM, which can be obtained from the Transfac or Jaspar databases. A value for each position was calculated based on the PWM value for the specific base. Underflow and the trivial zero result (if zero occurs in the PWM) were avoided as indicated.

The primoData function is used to generate a table with information about potential transcription factor binding sites discovered by searching promoters for matches to position weight matrices. The function is based on a previously published algorithm [29], which was implemented with some modifications in C++ (as PRIMO: PRomoter Integration in Microarray result Organization) [30] and in R for the pcaGoPromoter package in the present work. Fig. 1 illustrates the search algorithm for determining transcription factor binding sites using position weight matrices. The threshold score is calculated for each position weight matrix as the threshold that generates hits in a given percentage of all the promoters with a default of 10%, which is suggested in the original description of the algorithm [29]. When the highest possible score for position weight matrix identify more binding sites than 10% of all promoters, the highest possible threshold is chosen. The selection of the threshold for reporting a hit is thus based on the distribution of scores for a given position weight matrix in the promoter set being used. Other strategies for threshold selection based on e.g. a core motif [31] or motif conservation across species [32] have also been described in the literature. The primoData function is thus a tool for inclusion of custom promoter sets in the analysis. It takes as inputs two arguments (promoters, matrices). The promoters argument is a list with two elements. The first element is a list of Refseq identifiers and the second list element is a list of promoter sequences. The R command

Promoters <- pcaGoPromoters:::primoData.getPromoter( filename )

loads promoter sequences from a file in FASTA format into the promoters variable . The matrices argument is an R list of list elements. Each list element contain 3 data elements (baseId, name and pwm). baseId is a character vector with a base id, name is a character vector with the common name for the matrix, pwm is a position weight matrix with the base A,C,G,T in rows and the weights in columns. The primoData function is, however, only intended to be used by experts in bioinformatics because it requires a certain level of bioinformatics skills to obtain and format the input files from the public databases. In addition the function requires much processor time. The output of primoData is a data file to be used with the function primo, which is the function that actually joins the promoter analysis to the PCA analysis.

The R command:

myPrimoData <- primoData (promoters , fewMatrices)

calculates the myPrimoData object on custom promters and pwm matrices and the R command:

TFs <- primo( myLoadings , primoData  =  myPrimoData)

Calculates cis-element overrepresentation analysis on the set of probe set IDs (myLoadings) using the custom data.

To ease the use of the pcaGoPromoter package precalculated data files for promoters in the human, mouse and rat genomes are available on the project home pages (bioconductor and google.code). For these genomes, binding sites have been identified for promoter regions upstream of reference sequence mRNA transcripts (Refseq) [33]. We have defined the promoter region as 100 base pairs downstream and 1000 base pairs upstream of the 5′ end of each Refseq mRNA transcript, for a total of 1100 base pairs.

The input for primo is an object of class ExpressionSet. Alternatively a vector with either Affymetrix probe set IDs, gene symbols or Entrez IDs can be used in which case information about the organism is required and entered as the “org” argument. An option allows for the selection of multiple test correction using the argument p.adjust.method with the default being the false discovery rate. The result is a list of possible transcription factor binding sites that are either over- or underrepresented.

### Data – serum stimulation of a human monocyte cell line

To illustrate the use of pcaGoPromoter, including the rationale behind the analysis strategy, an experiment was designed and conducted. A serum-starved human monocyte cell line (ATCC Number: CRL-9853) was stimulated by serum in the presence (10 nM) or absence of the specific Erk-1/2 inhibitor, U0126 [34]. Twenty-two hours after serum addition, cells were harvested. RNA was extracted, and gene expression was analyzed by Affymetrix Human Genome U133 plus 2.0 arrays. Thirteen experiments were performed: five control experiments (serum-starved), three serum-stimulated, and five serum-stimulated in the presence of the inhibitor U0126. The addition of serum and inhibitor represented experimental manipulations, and these steps should be reflected in the independent effects identifiable in the principal components. Serum response is related to cell cycle progression [35], which in turn is regulated by E2F transcription factors [36]. Members of the Ets and Elk transcription factor families are the immediate early downstream nuclear targets of Erk-1/2 signaling [37], whereas activation of E2F transcription factors is a later event. Thus, it was expected that three groups of experiments would be discernible in the principal component analysis. Cell cycle progression should be reflected in the gene ontology analysis, and the Ets, ELK and E2F transcription factors were predicted to be revealed in the PRIMO analysis. Therefore, this experiment is well-suited to illustrate the functional interpretation of principal component score plot axes using overrepresentation analysis of annotation terms and predicted transcription factor binding sites. Data from the serum stimulation experiment are available at the gene expression omnibus under the accession number GSE27071 (http://www.ncbi.nlm.nih.gov/geo/query/acc.cgi?token=nvydbmmukoikora&acc=GSE27071).

## Results and Discussion

### Functional interpretation of monocyte serum response

The Affymetrix CEL files were read into R using the affy package followed by calculation of a normalized gene expression measure for each probe set ID using rma.

library(affy)

chipdata <- ReadAffy()

chipdataRMA <- rma(chipdata)

Load the pcaGoPromoter package.

library(pcaGoPromoter)

Do everything in one command (“groups” annotate experiments into classes in the plot. The variable is predefined to contain the classes: “control”, “serumInhib” and “serumOnly”):

pcaInfoPlot(chipdataRMA, groups = groups)

**Figure 2:**
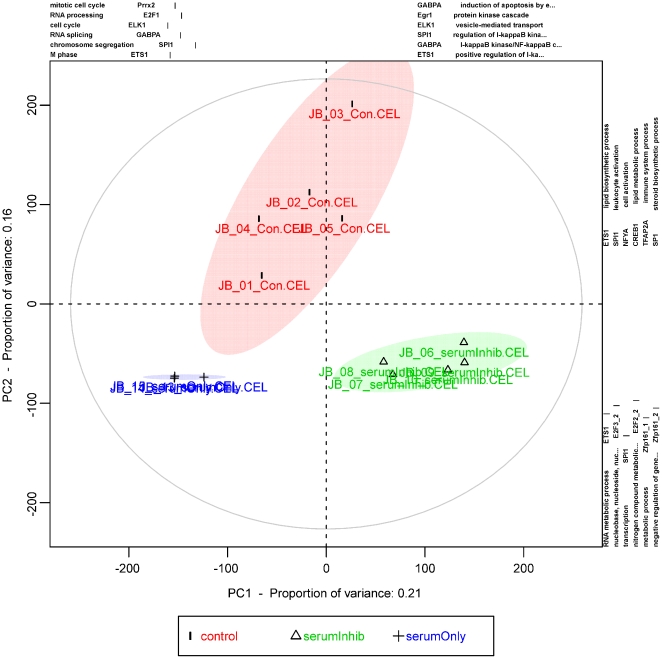
Principal component analysis score plot using pcaInfoPlot(). This plot shows the output from the function pcaInfoPlot(). This function makes a principal component analysis score plot and applies functional annotation to the axis. The plot shows the experiments of the three experimental groups (control, serum only and serum with inhibitor) separated into three clusters. The 1^st^ principal component (PC1), which contained 21% of the variance, shows the differences in gene expression caused by the inhibitor and the serum. The serum-only group (serumOnly) is in the most negative direction. The control group is in the middle. The serum with inhibitor (serumInhib) group is in the positive direction. The 2^nd^ principal component (PC2), which contained 16% of the variance, shows the effect of the added serum. The control group is in the positive direction, and the serum-supplemented groups (serumOnly and serumInhib) are in the negative direction. Each axis is functionally annotated with the five most significant GO terms (biological processes) and the five most significant overrepresented predicted transcription factor binding sites.

The resulting score plot (Fig. 2) displays the first two principal components. The experiments are colored according to the grouping vector, and a shaded ellipse marks the 95% confidence interval for the class. The experiments were grouped in clusters as expected: top (control), bottom left (serum only) and bottom right (serum with inhibitor). The three groups were separated along the 1^st^ principal component axis (x-axis), which explained 21% of the variance. This axis illustrates the portion of the serum effect influenced by the Erk-1/2 inhibitor. The control group was separated from the others along the 2^nd^ principal component axis (y-axis), which explained 16% of the variance. This axis illustrates the portion of the serum effect that is independent of the Erk-1/2 inhibitor. The axes were annotated with the top overrepresented GO terms and predicted transcription factor binding sites. The cell cycle progression was reflected in the negative direction of the 1^st^ principal component axis. Involvement of E2F and Ets transcription factors was predicted, because binding sites in gene promoters influence this direction of the axis.

The pcaInfoPlot function is designed to perform the key calculations required for functional interpretations of the PCA. The underlying calculations can be conducted individually with possibilities for choosing additional options as explained in the following.

Run PCA:

pcaObj <- pca(chipdataRMA)

The probe set IDs on the GeneChip can now be ranked according to their effect on the projection of the experiments into the new subspace defined by the 1^st^ and 2^nd^ principal component. The function getRankedProbeIds generates a ranked list of the probe set IDs that mostly contribute for placing experiments along the chosen principal component, here set by the argument “pc”:

probesPC1neg <- getRankedProbeIds(pcaObj, pc = 1, decreasing = FALSE)[1∶1365]

The number of probe set IDs chosen (1365) constitutes 1365/54613 = 2.5% of the total number of probe set IDs on the HG-U133 Plus 2.0 array.

The probes associated with the negative direction of the first principal component axis can now be interpreted in terms of biological processes:

GOtreeObj <- GOtree(probesPC1neg)

GOtree returns an object that lists all the GO terms with one or more genes, the total number of genes found for the term and a p-value calculated using an exact binominal test (binom.test(x, n, p)).

x: number of successes (number of probes in the extreme loading list having the specified GO term)

n: number of experiments (the total number of probes having the specified GO term)

p: the hypothesized probability of success (number of probes in the extreme loading list/total number of genes used in expression analysis)

Fisher's exact test for proportions can be used (option: significance Method = “fisher”)

**Figure 3:**
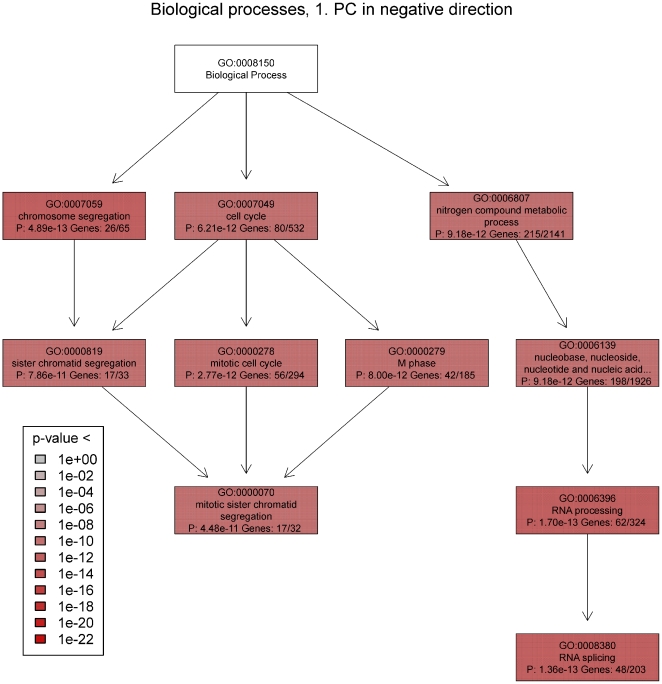
Negative direction PC1 gene ontology tree using 1365 probes. Significantly overrepresented gene ontology terms (biological processes) in the negative direction of the 1^st^ principal component using 2.5% of the most important probes were used to draw a tree graph. The gene ontology (GO) tree starts with the top term ‘biological process’ and then splits out into more specific terms. Box color indicates the p-value range. Each box contains the name of the process, the GO term number, the p-value, the number of genes in the subset and the total number of genes, which are annotated using this term. The GO tree splits into two major branches. The upper branch indicates cell division by mitosis, and the lower branch indicates mRNA processing.

Using the command plot(GOtreeObj) plots a tree view (Fig. 3) of the relations between the GO terms.

As seen in the pcaInfoPlot (Fig. 2), this analysis identified the two major branches in the GOtree of overrepresented biological processes. One branch was related to the cell cycle, and the other was related to RNA metabolism. The final leaf on the cell cycle branch in the GO tree (Fig. 3) was the term “mitotic sister chromatid segregation”. Thus, the 2.5% of genes with the most negative loadings in the 1^st^ principal component have an overrepresentation of annotation terms related to progression in the cell cycle. This was as expected for the “serum only” experiments, which were projected towards negative values of the 1^st^ principal component. Moreover, it can be hypothesized that the “serum inhibitor” experiments were also inhibited in cell cycle progression. This hypothesis requires experimental validation, e.g., DNA synthesis measurements using labeled nucleosides. It should be noted that the terms “negative” and “positive” only refer to the signs of the loadings following the PCA. The terms are thus purely mathematical and have no biological meaning.

Interestingly, GO overrepresentation analyses of the positive PC2 direction revealed overrepresentation of the term “cholesterol biosynthetic process” (p = 3.86×10^−5^). The function GOtreeHits found eight genes in the PC2 positive loadings annotated with this term. The probes interrogate genes involved in cholesterol synthesis (Table 1), a process that may be up-regulated in serum- starved cells due to a lack of cholesterol to provide lipoproteins (e.g., low-density lipoprotein; LDL) in the serum-free medium. Strict feedback control of cellular cholesterol biosynthesis is well-known [38], but changes in the expression patterns of genes involved in cholesterol biosynthesis were unexpected in the present serum stimulation experiment, which focused on the cell cycle and MAP kinase activation. However, the literature does provide support for serum starvation as an inducing stimulus for cholesterol synthesis [39]. This demonstrates that functional interpretation of score plot axes can yield useful insights into cellular processes.

**Table 1:** Probe set IDs annotated with GO 6695: “cholesterol biosynthetic process”.

Probe Set ID	Gene Symbol	Gene Title
201791_s_at	DHCR7	7-dehydrocholesterol reductase
200862_at	DHCR24	24-dehydrocholesterol reductase
203027_s_at	MVD	mevalonate (diphospho) decarboxylase
209279_s_at	NSDHL	NAD(P) dependent steroid dehydrogenase-like
202245_at	LSS	lanosterol synthase (2,3-oxidosqualene-lanosterol cyclase)
201275_at	FDPS	farnesyl diphosphate synthase (farnesyl pyrophosphate synthetase, dimethylallyltranstransferase, geranyltranstransferase)
211113_s_at	ABCG1	ATP-binding cassette, sub-family G (WHITE), member 1
200642_at	SOD1	superoxide dismutase 1, soluble (amyotrophic lateral sclerosis 1 (adult))

Overrepresented transcription factor binding sites in the promoters of genes defined by the probe set IDs with the 2.5% most extreme negative loadings for the 1^st^ principal component were found using the function primo:

primoRes <- primo(probesPC1neg)

The result “primoRes” contained two lists, overRepresented and underRepresented. Each list holds the respective transcription factor position weight matrix with p-values for over- and underrepresentation calculation using Fisher's exact test for proportions. Table 2 shows position weight matrices with overrepresentation hits in gene promoters (probe set IDs) with extreme (2.5%) positive or negative loadings. The list of position weight matrix hits for promoters associated with probe set IDs with the most negative loadings has three matrices for E2F transcription factors, whereas E2F position weight matrix hits were not found in the promoters associated with the probe set IDs with the most positive loadings in the 1^st^ principal component. This was as expected because the probe set IDs with the most negative loadings of the 1^st^ principal component represent cell cycle progression with activated E2F responsive promoters. Position weight matrix hits for the immediate downstream targets of MAP kinase activation, Ets and Elk transcription factors, are overrepresented in both directions of the 1^st^ principal component. The interpretation is that different Ets and Elk transcription factor targets are activated by serum in the absence and presence of the Erk-1/2 inhibitor.

**Table 2:** Overrepresentation analysis for predicted transcription factor binding sites using Primo on the 1^st^ principal component.

PC1 negative direction				
Matrix ID	Length	Name	Raw p-value	FDR
MA0098	6	ETS1	6,30E-16	6,17E-14
MA0080	6	SPI1	1,18E-13	1,16E-11
MA0062	10	GABPA	5,02E-09	4,92E-07
MA0024	8	E2F1	1,75E-05	1,72E-03
MA0028	10	ELK1	3,63E-05	3,56E-03
MA0076	9	ELK4	4,39E-04	4,30E-02
MA0075	5	Prrx2	6,74E-04	6,61E-02
MA0131	10	MIZF	3,43E-03	3,36E-01
MA0060	16	NFYA	4,19E-03	4,11E-01
PB0008	15	E2F2_1	4,19E-03	4,11E-01
PB0009	15	E2F3_1	1,96E-02	1,92E+00
PB0020	17	Gabpa_1	1,51E-01	1,48E+01
PB0027	17	Gmeb1_1	1,51E-01	1,48E+01
MA0004	6	Arnt	1,64E-01	1,60E+01
MA0104	6	Mycn	1,64E-01	1,60E+01
PB0095	16	Zfp161_1	6,05E-01	5,93E+01
PB0179	15	Sp100_2	1,02E+00	1,00E+02
MA0151	6	ARID3A	1,16E+00	1,14E+02
MA0006	6	Arnt::Ahr	1,49E+00	1,46E+02
MA0062	11	GABPA	1,74E+00	1,70E+02
PB0164	17	Smad3_2	6,08E+00	5,96E+02
MA0058	10	MAX	6,54E+00	6,41E+02
PB0108	14	Atf1_2	4,95E+01	4,85E+03
MA0259	8	HIF1A::ARNT	8,56E+01	8,39E+03

PC1 PositiveMatrix ID	Length	Name	Raw p-value	FDR
MA0098	6	ETS1	3,23E-18	5,36E-16
MA0080	6	SPI1	7,68E-14	1,27E-11
MA0062	11	GABPA	1,93E-13	3,21E-11
MA0028	10	ELK1	1,68E-07	2,79E-05
MA0076	9	ELK4	1,77E-07	2,93E-05
MA0062	10	GABPA	5,86E-07	9,73E-05
MA0162	11	Egr1	9,51E-07	1,58E-04
PB0020	17	Gabpa_1	3,35E-04	5,55E-02
MA0039	10	Klf4	5,11E-02	8,48E+00
MA0146	14	Zfx	4,96E-01	8,23E+01
MA0079	10	SP1	8,17E-01	1,36E+02
PB0039	16	Klf7_1	1,39E+00	2,31E+02
PB0011	15	Ehf_1	3,06E+00	5,09E+02
PB0189	14	Tcfap2a_2	8,19E+00	1,36E+03
MA0067	8	Pax2	1,17E+01	1,95E+03
PB0010	14	Egr1_1	2,65E+01	4,39E+03
PB0127	17	Gata6_2	4,10E+01	6,81E+03
MA0079	10	SP1	4,38E+01	7,27E+03

The probe set IDs joined to promoter hits for position weight matrices can be retrieved using the function primoHits as follows:

probeIdsE2F <- primoHits(probesPC1neg, id =  “9262”)

This function generates the list of probe set IDs associated with promoter hits for the E2F position weight matrix with the Jasper accession number MA0024 and ID 9252. A list of gene names can be retrieved using the mget function from the AnnotationDBI package and the hgu133plus2.db package:

geneNamesMA0024 <- mget(probeidsE2F, “hgu133plus2GENENAME”)

Among the resulting hits is proliferating cell nuclear antigen (PCNA), which is a well-known component of the DNA replication fork (for a review see [40]). Moreover, a functional E2F-binding site has been demonstrated in its promoter [41].

Predicted binding sites for proteins of the NF-κB transcription factor complex (c-REL (pwm MA0107) and NF-kB (pwm MA0061)) were also overrepresented in the gene promoters (probe set IDs) with extreme positive loadings for the 1^st^ principal component. This correlated with the overrepresentation of the GO term “regulation of I-kappaB kinase/NF-kappaB cascade” in the same direction of the 1^st^ principal component (Fig. 2). The interpretation is that the combined inhibitor and serum treatment led to NF-κB activation in the monocyte cell line. This is another example of an interesting result that is somewhat novel with respect to NF-κB activation. However, NF-κB inhibition by Erk-1/2 has been reported in endothelial cells [42] and again demonstrates the ability of our method to find and interpret biologically-relevant gene expression changes.

#### Relationship between loadings, variance and gene expression patterns

The first two principal components of the PCA (Fig. 2) explain 37% (21%+16%) of the variance in the original data. The variance in gene expression data can be interpreted in terms of gene expression patterns, which is a convenient way of interpreting the variance in gene expression analyses.

**Figure 4:**
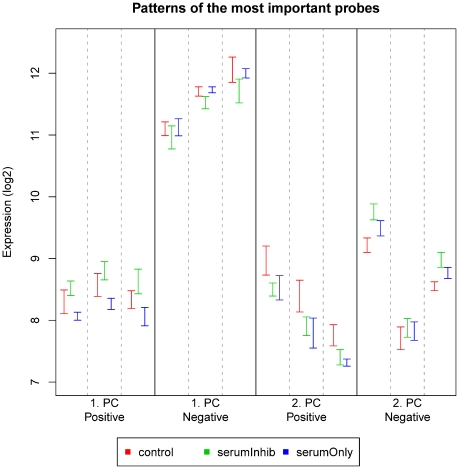
The three most important PC1 and PC2 probes in both the positive and negative directions. The probes have been selected by sorting the loadings from the PCA. The confidence intervals for the mean of each group (control, serumInhib and serumOnly) are plotted for each probe set ID.

Fig. 4 shows the gene expression measurements of the three probe set IDs with the most negative or positive loadings in the 1^st^ and 2^nd^ principal component. The probe set IDs with the highest positive loadings had expression patterns with the highest expression levels in the serum + inhibitor group for the 1^st^ principal component. The control group had an intermediate expression level, and the serum only group had the lowest expression level. For the probe set IDs with most influence on the negative direction of the 1^st^ principal component, the reverse was true. Likewise, probe set IDs with high or low expression in the control group relative to the two serum groups (with or without inhibitor) defined the positive or negative direction of the 2^nd^ principal component.

Thus, almost 40% of the variance in the original data was due to the four distinct gene expression patterns seen in Fig. 4.

#### Self-contained test for GO term overrepresentation

The test strategy used to calculate GO term overrepresentation is a competitive test strategy [26]. It depends on the total number of genes interrogated on the DNA chip, which include genes with functions unrelated to those of the genes with a particular GO term. Alternatively, a self-contained test strategy [26] that depends only on the genes with a particular joined GO term may be applied. Such a strategy is possible if an absolute value (e.g., a p-value) is used to determine if a gene is differentially expressed.

The GOtree() function can be used in a self-contained test.

As an example, the GO terms overrepresented in genes with increased expression in the serum-only samples compared with the control samples can be calculated in a self-contained test. First, genes with higher expression in serum-only samples are calculated using the t.test function in R. The resulting p-values are corrected for multiple tests by the false discovery rate method using p.adjust [43] and the probe set IDs with corrected p-values below the significance level subsequently stored in the variable selfcontained.

Then GOtree() is used with the binomAlpha argument set (p-value = 0.05):

GOselfcontained <- GOtree(selfcontained, binomAlpha = 0.05)

Table 3 shows the results for the comparison between the serum-only group and the controls. Mitosis and other terms related to cell cycle progression were overrepresented. This result is comparable to the GO analysis of the negative direction of the 1^st^ principal component (Fig. 2).

**Table 3:** Selfcontained test for overrepresentation (p<0.001) of GO terms in genes with higher expression in serum stimulated cells compared to controls (FDR<0.05).

GOid	Genes with term in list	Total number of genes with term	P-value	GOterm
GO:0007049	69	481	1,18E-11	cell cycle
GO:0022403	44	237	5,37E-11	cell cycle phase
GO:0022402	54	360	5,12E-10	cell cycle process
GO:0000278	45	281	2,40E-09	mitotic cell cycle
GO:0000279	33	163	2,40E-09	M phase
GO:0000280	26	108	4,65E-09	nuclear division
GO:0007067	26	108	4,65E-09	mitosis
GO:0000087	26	111	6,61E-09	M phase of mitotic cell cycle
GO:0034984	37	212	6,61E-09	cellular response to DNA damage stimulus
GO:0048285	26	111	6,61E-09	organelle fission
GO:0006259	49	353	2,47E-08	DNA metabolic process
GO:0006974	37	230	6,08E-08	response to DNA damage stimulus
GO:0007059	16	51	2,60E-07	chromosome segregation
GO:0033554	45	333	2,60E-07	cellular response to stress
GO:0006260	28	153	3,28E-07	DNA replication
GO:0051726	33	205	3,81E-07	regulation of cell cycle
GO:0007346	22	103	7,31E-07	regulation of mitotic cell cycle
GO:0006281	29	173	1,15E-06	DNA repair
GO:0006297	9	17	3,09E-06	nucleotide-excision repair, DNA gap filling
GO:0000075	16	71	3,12E-05	cell cycle checkpoint
GO:0051716	49	460	6,37E-05	cellular response to stimulus
GO:0000070	9	27	3,25E-04	mitotic sister chromatid segregation
GO:0065004	11	42	3,81E-04	protein-DNA complex assembly
GO:0000819	9	28	4,19E-04	sister chromatid segregation
GO:0006323	13	61	5,93E-04	DNA packaging
GO:0051276	31	266	8,76E-04	chromosome organization

### Parameter selection

As explained in the preceding sections the sign and the magnitude of the loadings indicate the importance of a probe set ID for a given principal component. Thus to join a functional interpretation to a principal component the probe set IDs with the highest absolute loadings with either positive og negative signs are retrieved and analyzed for overrepresentation of GO terms in the annotation or for overrepresentation of potential transcription factor binding sites in the corresponding promoters. A facing issue is the decision about at which magnitude of loading to set the cut-off. For the serum stimulation example we have used the 2.5% of the probe set ID variables with the highest absolute loadings in both PC directions. The 2.5% cut-off was chosen here as it yielded biological sound interpretations of gene expression data in previous analysis (e.g. [12], [14]). However, in other settings it may be useful to be able to change the cut-off and to study the effect of changing it.

**Figure 5:**
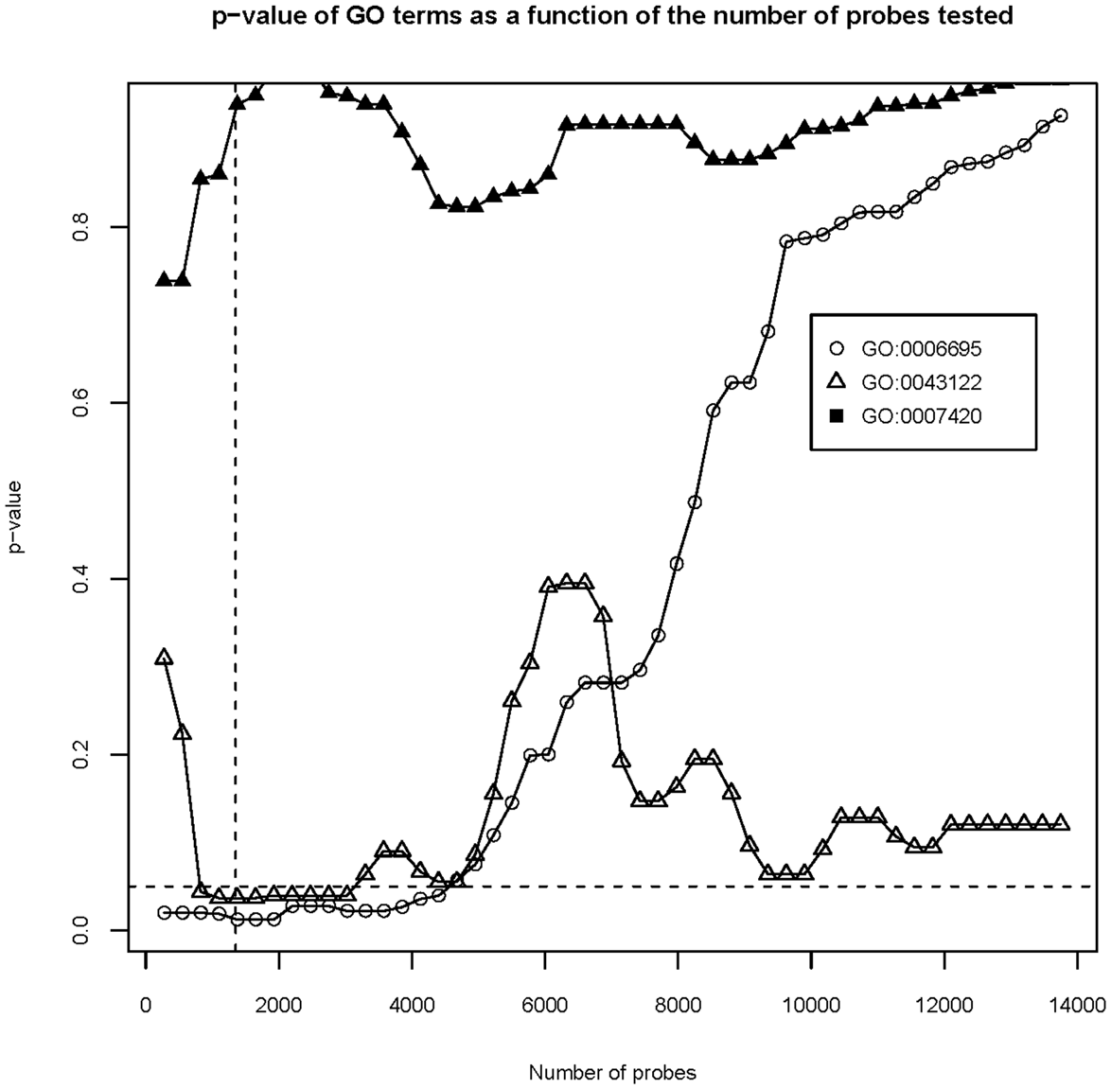
The Importance of loading cut-off for inclusion of probe set IDs in GO term annotation analysis. The probe set IDs were sorted after loadings for the first principal component (GO:0043122 and GO:0007420) and second principal component (GO:0006695) following the PCA of the serum stimulation data (Figure 2). Overrepresentation analysis for the three terms was repeated for different cut-off values between 0.5% and 25% of probe set IDs with highest loadings. Shown are the resulting p-values. The vertical dotted line indicates the top 2.5% probe set IDs with highest loadings. This is the cut-off used in the text and as the default in the pcaInfoPlot function. The horizontal dotted line indicates the 0.05 significance level.

We first consider the significant GO term GO:0006695 (cholesterol biosynthetic process) which was found overrepresented in probe set IDs with the 2.5% highest loadings in PC2. Fig. 5 depicts the effect on the p-value of changing the fraction of probe set IDs included from the 0.5% highest loadings to the 25% highest loadings. It can be seen that the overrepresentation (p<0.05) of this term is observed already when 0.5% of the probe set IDs with the highest loadings are included and the overrepresentation is maintained until 8.5% of the probe set IDs with highest loadings are included. Further inclusion of probe set IDs leads to p-values above 0.05. This is due to inclusion of probe set IDs not annotated with the specific term in question. In fact only 16 genes on the chip are annotated with this term (GO:0006695) and 8 of these are found in the top 2.5% of probe Set IDs with highest loadings. Clearly the number of probe set IDs annotated with a given term will influence the outcome of the parameter selection. We therefore now consider the term GO:0043122 (“regulation of I-kappaB kinase/NF-kappaB cascade”), which is annotated to 105 probe set IDs on the chip. This term is overrepresented in the interval from 1.5% to 5.5% included probe set IDs. For this term the changes of the p-values are not monotone for increasing values of the cut-off (Figure 5). This is due to groups of probe set IDs in the interval between 5.5% to 25% that are annotated to this term having similar loadings followed by groups of probe set IDs not annotated to this term.

The term GO:0007420 (“Brain development”) is annotated to 80 probe set IDs on the chip and was not found overrepresented in the analysis. The calculated p-value for this term remained high irrespective of the fraction of probe set IDs included (Fig. 5). In the selection of probe set IDs for the overrepresentation analysis we could have weighted the probe set IDs by the PCA-rank instead of letting all included probe set IDs contribute equally to the test statistics. However, the results depicted in Figure 5 suggest that taking rank into account would only have minor effect. Thus both significant terms remain significant over a relatively broad interval of included probe set IDs. The main conclusion is that an interval between 1.5% and 5% of included probe set IDs yields robust results. Similar findings were found for the primo analysis.

### Robustness under data perturbation

Cross-validation is a direct way to judge the robustness of the PCA and the joined functional interpretations of the PC axes. This can be achieved using the functions GOtreeWithLeaveOut and primoWithLeaveOneOut. Thus, a fraction of experiments are left out, and the PCA model builds using a data set with a reduced number of experiments. This process is repeated until all samples have been left out. The default is leave-one-out, but a fraction of the samples to be left out can be given as an argument (e.g., leaveOut = 0.1 results in the omission of 10% of the samples in each run). For both functions, only GOterms (for GOtreeWithleaveoneOut) or PWMs (for primoWithLeaveOneOut) that are present in all runs are retrieved for subsequent ranking after p-value. Thus, GO terms or PWMs that are only found in some of the cross-validation runs are not present in the final list.

The command line:

GOtreePC2poscv <- GOtreeWithLeaveOut(exprsData, pc = 2, decreasing = TRUE)

calculates overrepresented GO terms in the positive direction of the 2^nd^ principal component using leave-one-out cross-validation. The results (Table 4) show that GO terms related to sterol metabolism were consistently overrepresented in the positive direction of the 2^nd^ principal component.

**Table 4:** Overrepresentation (p<0.05) of GO terms in the annotation of genes defining the positive direction of PC2 calculated using leave-one-out cross-validation.

GOid	p-value	Total number of genes with term	GOterm
GO:0008610	0,004	185	lipid biosynthetic process
GO:0002376	0,008	616	immune system process
GO:0008284	0,010	221	positive regulation of cell proliferation
GO:0045321	0,010	172	leukocyte activation
GO:0006629	0,011	480	lipid metabolic process
GO:0016126	0,013	14	sterol biosynthetic process
GO:0008202	0,013	117	steroid metabolic process
GO:0001775	0,017	199	cell activation
GO:0008652	0,017	15	cellular amino acid biosynthetic process
GO:0009309	0,019	31	amine biosynthetic process
GO:0042127	0,019	447	regulation of cell proliferation
GO:0019752	0,024	297	carboxylic acid metabolic process
GO:0006950	0,026	984	response to stress
GO:0006694	0,026	49	steroid biosynthetic process
GO:0006082	0,027	304	organic acid metabolic process
GO:0042180	0,027	304	cellular ketone metabolic process
GO:0006520	0,027	98	cellular amino acid metabolic process
GO:0048659	0,028	19	smooth muscle cell proliferation
GO:0033138	0,031	11	positive regulation of peptidyl-serine phosphorylation
GO:0016477	0,032	214	cell migration
GO:0006695	0,034	12	cholesterol biosynthetic process
GO:0008203	0,034	41	cholesterol metabolic process
GO:0006928	0,043	346	cellular component movement
GO:0030032	0,044	6	lamellipodium assembly
GO:0006066	0,047	212	alcohol metabolic process
GO:0048870	0,048	235	cell motility

### Relationship to other annotation analysis strategies

Classically the biplot [44] is used in PCA and related multivariate analysis methods for displaying the relationship between variables and experiments in the same 2D-plot. Our analysis strategy focuses, however, on the PC axes and is only equivalent to a biplot analysis when the experiments are clearly grouped and positioned close to the axes. The advantage of our analysis strategy is that the axes can be interpreted in relation to function and regulatory mechanisms even in the case where the experiments are not clearly grouped in the plot. We believe that our method of interpreting the axes is intuitive to biologists who are not *a priori* experts in bioinformatics or biostatistics. Advanced users interested in higher-level analysis of the link between annotation and genome-wide gene expression data are referred to [45], [46], [47], [48].

The PcaGoPromoter analysis strategy relies on overrepresentation analysis. An alternative strategy would be to form an aggregate score for a gene set defined by a GO term or a transcription factor binding site. A very popular method depending on aggregate scores is the gene set enrichment analysis (GSEA; [49]). One theoretical advantage of methods depending on aggregate scores is that they only rely on the information gathered from the genes included in the gene set. In the standard use of pcaGopromoter, probe set IDs not annotated with a given term contribute to the calculation of the p-value for overrepresentation. To give the user the ability to calculate overrepresentation which is only dependent on the probe set IDs annotated with a given GO term, the GOtree function was supplemented with the self-contained test option (see above).

### Conclusions

The R package pcaGoPromoter provides a collection of tools for the analysis of gene expression data obtained from any genome-wide expression analysis platform supporting either of Affymetrix probe set IDs, gene symbols or Entrez IDs as probe identifiers. It was developed in the statistical environment R. The package pcaGoPromoter provides functions to give an overview of the data by PCA, functional interpretation by gene ontology terms (biological processes), and an indication of the involvement of specific transcription factors. In the present setup, a serum stimulation experiment with a monocyte cell line was used for illustrative purposes. In addition to the expected results, the pcaGoPromoter analysis also revealed unexpected and interesting results when applied to the serum stimulation data, e.g., an indication of cholesterol synthesis in serum-starved cells and NF-κB activation in cells treated with both serum and Erk1/2 map kinase inhibitor. This directly demonstrates how the pcaGoPromoter package can be used to direct attention towards relevant biological issues in various genome-wide gene expression analyses in the future.

### Web site access

A pcaGopromoter online version providing access to the most important plot functions is available at http://gastro.sund.ku.dk/brew/pcaGoPromoter.html .The serum stimulation experiment used for calculations in this presentation is available as an example. In addition the user can upload data for analysis. The uploaded data should be either a zipped CEL file (with the Affymetrix platform) or a csv table for other formats. The extension R package can also be downloaded and installed locally.

### Availability

**Project name:** pcaGoPromoter

**Project home page:** http://gastro.sund.ku.dk/brew/pcaGoPromoter.html

**Public repositories:**

https://code.google.com/p/pcagopromoter/downloads/list

http://www.bioconductor.org/packages/2.10/bioc/html/pcaGoPromoter.html

**Operating system(s):** Linux, Windows, Mac OS X

**Programming language:** The R statistical environment

**Other requirements:** R version 2.10 or higher, Bioconductor 2.x

**License:** GNU GLP3

**Any restrictions to use by non-academics:** None
